# Does the athletes’ body shape the athletes’ mind? A few ideas on athletes’ mental rotation performance. Commentary on Jansen and Lehmann

**DOI:** 10.2478/v10053-008-0136-7

**Published:** 2013-06-17

**Authors:** Thomas Heinen

**Affiliations:** Institute of Sport Science, University of Hildesheim, Germany

**Keywords:** embodiment, mental simulation, congruency effect

## Abstract

Athletes exhibit differences in perceptual-cognitive abilities when compared to non-athletes. Recent theoretical developments focus on the role of the athletes’ body in perceptual-cognitive tasks such as mental rotation tasks. It is assumed that the degree to which stimuli in mental rotation tasks can be embodied facilitates the mental rotation process. The implications of this assumption are discussed and ideas for future research are presented.

## Commentary

In their article, Jansen and Lehmann (2013) proposed that there exists a relationship between participants’ sport-specific experience and their mental rotation performance. In their study, gymnasts and soccer players were asked to solve a mental rotation task incorporating three-dimensional cube and human figures. Results revealed that gymnasts exhibited a higher test performance when compared to non-athletes, and mental rotation performance was in general better for pictures of human figures than for pictures of cubed figures. The authors argued that mental rotation performance might be selectively affected by enhanced physical activity in one sport. However, gymnastics and soccer place different demands on athletes, which in turn may lead to considerably different sensory-motor experiences over years of practice. Thus, the question arises if differences in mental rotation performance are merely based on enhanced physical activity in one sport or if they are potentially grounded in the individual shape and specificity of athletes’ bodily systems (such as, e.g., the neuro-muscular system)?

Wexler, Kosslyn, and Berthoz (1998) have already hypothesized that “mental rotation is a covert simulation of motor rotation” (p. 78), highlighting the potential role of the motor system in mental rotation tasks in particular, thereby supporting the assumption that the motor system plays an important role in (motor) imagery tasks in general (Jeannerod, 2001; Munzert, Lorey, & Zentgraf, 2009; Richter et al., 2000). It is furthermore argued that the perceptual-cognitive abilities of athletes are significantly shaped by the massive experience they have accumulated over the years of practice in planning and executing self-produced activities (Blake & Shiffrar, 2007; O’Regan & Noë, 2001). This experience usually goes along with adaptations in the various body systems. Taking into account the mental rotation task in Jansen and Lehmanns’ (2013) article together with the results of their study, it seems necessary to more closely focus on the potential role of athletes’ body in its perceptual and cognitive processes (Gibbs, 2006). Just as computers with different hard- and software configuration may produce different results in processing data (such as images or video files), it seems inappropriate to assume that athletes who are experts in different sports process sensory stimuli in the same way.

The relevant assumption for mental rotation here is quite straightforward: If athletes can *embody* the stimulus (or characteristics of it) in a mental (rotation) task, then this would facilitate the mental (rotation) process (Amorim, Isableu, & Jarraya, 2006). This embodiment process may however depend on factors such as the structure and function of the athletes’ body (as well as age and gender), the athletes’ body position and body posture, the athletes’ state of motion, current actions and sensory input, the athletes’ bodily states, as well as the athletes’ sensory and motor experience (e.g., Barsalou, 2008; Goldman & de Vignemont, 2009; Jacobs & Shiffrar, 2005; Janczyk, Pfister, Crognale, & Kunde, 2012; Lenggenhager, Lopez, & Blanke, 2008; Proffitt, Stefanucci, Banton, & Epstein, 2003; Teachman, Stefanucci, Clerkin, Cody, & Proffitt, 2008; Witt, Linkenauger, Bakdash, & Proffitt, 2008; Wohlschläger & Wohlschläger, 1998).

Empirical evidence is in line with the assumptions just mentioned. For instance, mental rotation is faster and more accurate when participants produce concurrent movements or postures that are compatible with and/or congruent to the mental rotation task (Amorim et al., 2006; Ionta, Fourkas, Fiorio, & Aglioti, 2007; Wohlschläger & Wohlschläger, 1998). Such a congruency effect is likely to occur when comparing, for example, gymnasts and soccer players in a mental rotation task that more strongly reflects gymnastic-specific postures and/or rotation demands than soccer-specific postures and/or rotation demands. Given that mental rotation performance strongly depends on the congruency between the bodily characteristics of the stimuli and the participant, the question arises if not only the stimuli (human figures vs. cube figures) but rather the congruency between stimuli and athletes’ bodily characteristics would be a better predictor for mental rotation performance in athletes from different sports when compared to non-athletes. In addition, the current work neglects participants’ whole-body rotations (e.g., pirouettes or twisting and non-twisting somersaults in gymnastics) as well as more complex and sport-specific concurrent movements (e.g., symmetrical or asymmetrical arm-, leg-, trunk-, or head-movements in martial arts) when performing mental rotation tasks. The question thus arises whether congruent body orientations and more complex whole-body rotations have similar effects on mental rotation as (rather simple) hand movements and poses (congruency effects). Furthermore, there is no evidence on the effect of subject’s actively performing whole-body rotations on mental rotation performance (concurrency effects). Additionally, proofs for the interaction of congruency and concurrency effects are limited to simple movements and need yet to be generalized for whole body rotations (e.g., mentally rotating an image of ones’ own hand whilst manually rotating ones’ own hand in the direction of the shortest rotation path in the mental rotation task).

Further research could possibly be realized by using a whole-body rotation device (human gyroscope) in combination with a virtual realityhelmet that would allow to independently manipulate congruency between athletes’ posture/body orientation and the stimuli’s posture/object orientation, athletes’ rotation activity, and athletes’ concurrent movements during the mental rotation task. Behavioral measures should be assessed together with neurophysiological measures in order to differentially analyze athletes’ choice responses and choice response durations in the mental rotation task (Magill, 2011), since the same response duration or the same choice response in two athletes from different sports could potentially be a product of different internal processes (e.g., Soichi, Kida, & Oda, 2001). Furthermore, variants of different mental rotation tasks should be applied, since they may have different outcomes depending on athletes’ type of sport and/or the type of sport that is reflected in the mental rotation stimuli (De Lange, Helmich, & Toni,2006; Jansen & Lehmann, 2013). The approaches just outlined could help to identify the mechanisms underlying the effects of congruency via active rotations, (in)congruent postures and stimulus similarity.

It is most desirable to close the gaps in our knowledge and thus to advance our concepts about spatial cognition, object recognition, and imitation. On a transfer level, this would be beneficial for our understanding of motor learning based on imitation and observation (Hodges & Williams, 2007), mental simulation (Faubert & Sidebottom, 2012), and physical guidance (Wulf, Shea, & Whitacre, 1998), and could contribute to the training of athletes from sports such as skydiving, scuba-diving, and climbing, where losses of spatial orientation can be life-threatening. Studies on the effects of motor learning on mental rotation performance are further warranted, since there is only very restricted empirical evidence on the effects of body-rotation training on mental rotation performance (Hecht, Vogt, & Prinz, 2001; Jansen, Titze, & Heil, 2009). The study of Jansen and Lehmann (2013) can be seen as one important step for significant future theoretical and methodical developments in the field of mental rotation in sports.
